# Day-old chicken quality and performance of broiler chickens from 3 different hatching systems

**DOI:** 10.1016/j.psj.2020.12.050

**Published:** 2020-12-23

**Authors:** Carol Souza da Silva, Roos Molenaar, Mona F. Giersberg, T. Bas Rodenburg, Johan W. van Riel, Kris De Baere, Iris Van Dosselaer, Bas Kemp, Henry van den Brand, Ingrid C. de Jong

**Affiliations:** ∗ Wageningen Livestock Research, Wageningen University and Research, Wageningen 6700 AH, The Netherlands; † Adaptation Physiology Group, Wageningen University and Research, Wageningen 6700 AH, The Netherlands; ‡ Animals in Science and Society, Department of Population Health Sciences, Faculty of Veterinary Medicine, Utrecht University, Utrecht 3508 TD, The Netherlands; § Experimental Poultry Centre, Province of Antwerp, Geel 2440, Belgium

**Keywords:** broiler, on-farm hatching, early nutrition, performance, breast myopathies

## Abstract

In on-farm hatching systems, eggs are transported at d 18 of incubation to the broiler farm, where chickens have immediate access to feed and water after hatching. In hatchery-fed systems, newly hatched chickens have immediate access to feed and water in the hatchery and are transported to the farm thereafter. Conventionally hatched chickens can remain without access to feed and water up to 72 h after hatching until placement on the farm. The current study compared day-old chicken quality, performance, and slaughter yield of broiler chickens that were on-farm hatched (**OH**), hatchery-fed (**HF**), or conventionally hatchery-hatched (**HH**). The experiment was performed in 6 rooms in 1 house. Each room contained 2 duplicate pens with approximately 1,155 chickens per pen; 2 rooms with each 2 duplicate pens were assigned to 1 treatment. The experiment was repeated during 3 consecutive production cycles. Chickens originated from young parent stock flocks. Results showed that HF and OH chickens were heavier and longer than HH chickens at day (**D**) 1. Relative weight of stomach and intestines were highest for OH chickens. The OH chickens had worse day-old chicken quality in terms of navel condition and red hocks than HH and HF chickens. Treatments did not differ in first wk and total mortality. From D0 until slaughter age, body weight was highest for OH, followed by HF and HH. Furthermore, carcass weight at slaughter age (D40) was highest for OH chickens, followed by HF and HH chickens. Breast fillets showed a higher incidence of white striping and wooden breast in HF and OH chickens compared with HH chickens. In conclusion, the current study showed that both OH and HF chickens of young parent flocks had better growth performance, which could explain the higher prevalence of breast myopathies, compared with HH. The worse day-old chicken quality for OH compared with HH and HF does not seem to affect first wk mortality and later life performance.

## Introduction

In a commercial hatchery, the majority of broiler chickens starts to hatch after 19 d of incubation. The length of the “hatch window” (period between first and last chicken that hatches) ranges between 24 and 36 h (Careghi et al., 2005; van de Ven et al., 2009). Standard practice in conventional hatcheries is that all chickens stay in the incubator until at least 510 h of incubation, after which they are collected and processed. This period, added to the time required for handling procedures (e.g., processing, selection of second grade chickens, sex determination, and vaccination) and transport to the broiler farm, can result in up to 72 h of feed and water deprivation for day-old chickens (Willemsen et al., 2010). Previous studies showed that posthatch feed and water deprivation longer than 36 h may impair organ development (de Jong et al., 2017) and particularly intestinal development (Bigot et al., 2003; Lamot et al., 2014), immunological development (Bar Shira et al., 2005; Panda et al., 2015), and capacity to withstand cold exposure (van den Brand et al., 2010) in the first wk of life. Moreover, it has been shown that prolonged duration of feed and water deprivation (≥36 h) results in a lower body weight and higher total mortality in broiler chickens up to 6 wk of age (de Jong et al., 2017). In addition to posthatch feed and water deprivation, conventionally hatched chickens are exposed to several environmental stressors during the perinatal period, such as disinfection, dust, and pathogen load (de Gouw et al., 2017), continuous darkness (Archer and Mench, 2014), noise levels, handling, and subsequent transportation (Hollemans et al., 2018). This can have long-term consequences for health and development of the chickens (Ericsson et al., 2016; Hedlund et al., 2019) and thereby also for their performance.

To prevent negative effects of posthatch feed withdrawal on broiler health, development, and performance, alternative hatching systems that allow immediate access to feed and water after hatching have been developed. In practice, early provision of feed and water is done by either hatching eggs on the broiler farm (on-farm hatching) or supplying feed and water in the hatchers (hatchery-fed) (Hollemans et al., 2018). In on-farm hatching systems, eggs are transported to the broiler farm at d 18 of incubation, where chickens have immediate access to feed and water posthatch. In a hatchery-fed system, newly hatched chickens receive feed and water in the hatchery and are transported to the farm in the baskets in which they hatch. Taken together, there might be an overall reduction in stressful events that occur in the early life of a chicken with on-farm hatching or hatchery feeding (Hollemans et al., 2018; de Jong et al., 2019), and this may potentially improve welfare (including health) and performance in later life. However, scientific evidence regarding the effects of these alternative hatching systems on broiler performance is still limited.

In recent studies, performance and welfare of broiler chickens that hatched conventionally or on-farm were compared, either under commercial (de Jong et al., 2019) or more controlled conditions (de Jong et al., 2020). These studies found worse navel and hock quality at d 0 but better welfare in terms of lower first wk and/or total mortality and less footpad dermatitis in on-farm hatched chickens compared with conventionally hatched chickens. However, regarding body weight, effects in prime flocks were only short term with higher body weights until 7 d (de Jong et al., 2019) or 21 d of age (de Jong et al., 2020) in on-farm hatched flocks compared with hatchery-hatched flocks. This indicates that long-term benefits of on-farm hatching on performance may be lacking, although this can also be influenced by other factors such as parent stock age or rearing management, which deserves further research. In addition, it is unclear how the provision of feed and water in the hatchery is comparable to on-farm hatching, as hatchery-fed chickens still experience transport to the broiler farm, which is an additional stressor compared with on-farm hatching.

Therefore, the aim of the present study was to compare day-old chicken quality and performance of broiler chickens that were conventionally hatched, with those that were fed in the hatchery or hatched on-farm. Chickens originating of a young parent stock (28–31 wks of age) were chosen, as these usually have a lower body weight and are more vulnerable in the early posthatch period (Weytjens et al., 1999; Vargas et al., 2009; Nangsuay et al., 2013) and may therefore benefit more from early feeding than chickens of a prime or old breeder flock. We hypothesized that the reduction of stressors with both on-farm hatching and hatchery feeding would result in improved performance in chickens from young parent stock as compared with conventional hatchery-hatching and that this effect would be largest in on-farm hatched chickens, as these were not subjected to transport.

## Materials and methods

The experiment was approved by the Institutional Animal Use and Care Committee of the Experimental Poultry Centre (Geel, Belgium; License number EC 2019001).

### Study Design, Hatching and Incubation Process, and Housing

The experiment was carried out at the Experimental Poultry Centre in Geel, Belgium, during 3 consecutive production cycles between May and October 2019. Three treatments were applied: conventional hatching at the hatchery without light, feed, or water in the hatcher (hatchery-hatched, **HH**), hatching at the hatchery with light exposure and provision of feed and water in the hatcher (hatchery-fed, **HF**), and on-farm hatching (**OH**) with light exposure, where feed and water were available after hatch and, in addition, transport of day-old chickens from hatchery to farm was not needed. The day at which the HH and HF chickens arrived from the hatchery at the research facility was, according to commercial practice, named “day (**D**) 0” for all the treatments.

For each production cycle, all eggs were incubated at a commercial hatchery (Lagerwey, Lunteren, The Netherlands) in 1 incubator (MicroClimer Setter 12, HatchTech B.V., Veenendaal, The Netherlands) with a maximum capacity of 84,480 eggs. Eggs were gradually heated in the incubator from the storage temperature to the incubator temperature within 22 h. The incubator temperature was gradually decreased throughout incubation to obtain an egg-shell temperature of 37.8°C, relative humidity was set at 75% and decreased gradually toward 30%, and CO_2_ concentration was maintained below 3,500 ppm (embryonic day [**E**] 0 to 18). At E18, all eggs were transferred to either hatching baskets or trays. Eggs were candled with a heartbeat system, and trays with eggs were randomly assigned to 1 of the treatments (HH, HF, or OH).

Hatchery-hatched chickens hatched according to standard commercial procedures; fertile eggs were transferred to regular hatching baskets (595 × 397 × 166 mm; maximum capacity of eggs per basket) and were exposed to disinfection at E18 and E19 with 200 ml of formaldehyde solution (37%). The HH chickens were collected from the incubators at 510 h of incubation (excluding the preheat time of 22 h), followed by standard hatchery procedures, including transportation on conveyor belts, selection of second grade chickens (e.g., small chicks, unable to stand straight up, or abnormalities; Lourens et al., 2005), and temporary storage in a chicken room. The HH chickens were transported to the experimental farm at D0. Transport time was 2.5 h, and total storage time and transport and did not exceed 4 h. The HH chickens did not have access to feed and water until placement in the pens on the experimental farm.

For the HF treatment, fertile eggs were transferred at D18 to the HatchCare system (HatchTech B.V.), consisting of cradles (673 × 580 × 166 mm; 90 eggs per cradle) with an overlay egg tray. Via one of the open spaces on the egg tray, newly hatched chickens were able to fall into the cradle with integrated feeding troughs on both sides of the cradle, containing 350 g of feed (prestarter, 2900 kcal/kg; raw protein 21%, raw fat 6%). Water was available from a drinking gutter on one side of the cradle above which a strip with LED lights was installed (272 lux at chicken level). The cradle contained a plastic grid floor where manure could fall through on a second plastic layer. Similar to the HH eggs, HF eggs were exposed to disinfection with formaldehyde solution (37%) at E18 and E19. To minimize handling, processing and selection of second grade HF chickens was done within the cradle at 516 h after the start of incubation. Chickens remained in the same cradle from E18 of incubation until arrival on the experimental farm. The HF chickens were transported to the experimental farm at D0 with a transport time of 2.5 h; total storage and transport time did not exceed 4 h. The HF chickens had access to feed that was left in the integrated feeding troughs during transportation.

For the OH treatment, 18-d incubated fertile eggs were transported on egg trays to the experimental farm, where the egg trays were placed in the X-Treck system (Vencomatic, Eersel, The Netherlands; de Jong et al., 2019, 2020) and hatched in the broiler house. The OH eggs were not exposed to disinfection between E18 and hatching. The X-Treck system consisted of 8 setter trays with 150 fertile eggs per pen that were placed on a suspended rail system 15 cm above a polypropylene belt, which was placed 30 cm above the floor. After emergence from the eggshell, chickens fell on the belt. After drying on the belt, they moved to the edge of the belt and fell on the litter, where feed and water were provided. Light was continuously on from E18 up to D0 to enable the chickens to find food and water after hatching. After hatching, the suspended rail system was lifted to the ceiling and trays with eggshells, and nonhatched eggs were removed from the house. Selection of second grade OH chickens was done in the pen by the animal caretakers (according to Lourens et al., 2005).

In each production cycle, the same 6 identical rooms were used with central heating and a separate climate control system per room. Each room had 2 equal pens (each pen measuring 6.0 × 9.4 m, separated by a wire mesh), and 1 treatment (HH, HF, or OH) was assigned to both pens in 1 room. This resulted in 2 replicate rooms (each with 2 duplicate pens) per treatment for each of the 3 consecutive production cycles. The 6 rooms were located adjacent to each other in 1 broiler house and connected by a central corridor. Pens had their own automated feeders and drinkers, enabling registration of feed and water intake at pen level. Each pen was equipped with 14 feeder pans distributed over the pen and 2 drinker lines with 84 nipples in total. Fresh wood shavings (1.5 kg/m^2^) as litter material were distributed before placement of the eggs, and in the OH pens, a small amount of litter material was distributed over the conveyor belts.

### Animals and Management

In total, 41,398 Ross 308 broiler chickens were used for the grow-out period. The broiler breeder flocks were 28, 29, and 31 wk of age for production cycles 1, 2, and 3, respectively. The HH chickens arrived from the hatchery and were placed in the morning of D0, whereas HF chickens were placed 6 h later than the HH chicks, that is in the afternoon of D0. On D0, 1,155 day-old chickens per pen were present in cycles 1 and 2, and 1,141-day-old chickens per pen were present in cycle 3 for all treatments. The final stocking density in all pens at slaughter weight did not exceed 42 kg/m^2^ according to legislation.

All chickens received spray vaccinations for infectious bronchitis and Newcastle disease at D0 (on the experimental farm) and D19 and Gumboro vaccination at D13. Additional feed was provided on chicken paper in all pens during the first days, starting at E18 for OH pens. A standard commercial four-phase feeding program was applied (Aveve, Merksem, Belgium), and both feed and water were provided *ad libitum*. For the starter diet, the energy content, raw protein (**RP**), and raw fat (**RF**) contents were 3,009 kcal/kg, 20.7%, and 6.4%, respectively. Dietary protein and fat contents of the other diets were grower 1 RP 20.7%, RF 7.3%; grower 2 RP 22.6%, RF 9.1%; finisher RP 23.7%, RF 10.2%. Whole wheat was added to the diet from D8 onward (5%) until slaughter age (40%). For the OH groups, the environmental temperature between E18 and D0 was based on measurements of eggshell temperature, recorded on E18 and E19. The eggshell temperature was maintained around 37.2°C. The average room temperature during E18 to D0 was 34°C with a relative humidity of 47%. The room temperature decreased from approximately 34°C at D0 (HH) to 19°C at D40. Temperature settings were adjusted based on chicken temperature measurements in the first wk of age, which resulted in 1°C lower room temperature for OH than for HH and HF in batch 1 and 1°C lower room temperature for OH and HF than for HH in batch 2 and 3. Rectal temperatures of all treatments ranged between 41.0 and 41.5°C in the first wk. On D0, 1 h of darkness was provided, which increased to 6 h of darkness from D6 onward; lights were on from 04:00 to 07:00 h, 08:00 to 20:00 h, and 21:00 to 00:00 h, except during the second production cycle. In that cycle, the light schedule was adjusted from D13 onward, so that lights were on from 01:00 to 12:00 h, 13:00 to 16:00 h, and 20:00 to 00:00 h to prevent heat stress during a heat wave. During the final 3 d before depopulation (D38–40), a 23L:1D schedule was applied (lights off from 00:00 h–01:00 h). Thinning was performed at D33 by taking out 280 broilers from each pen. The remainder of the broilers stayed until all pens were processed at D40. After cleaning and disinfection, a new cycle started after 2 to 3 wk with the placement of 18-d incubated eggs in the OH pens. Owing to a high mortality (primarily related to yolk sac infection) in the first wk of production cycle 3, all treatment groups were treated with Methoxasol 20 mg/100 ml (dosage: 33 mg/kg, from D3 until D6).

### Embryo Mortality and Hatchability of Fertile Eggs

To classify embryonic mortality, a breakout analysis of the nonhatched eggs was performed on 10 baskets (75 fertile eggs/basket) for the HH, on 10 baskets (90 fertile eggs/basket) for the HF, and on 32 trays (150 fertile eggs/egg tray) for the OH groups. For every nonhatched egg, a distinction was made between the stage of embryonic development: before internal pipping, internal pipping, external pipping, or alive. Malformations and malpositions were recorded in addition. Percentages of these distinctions were calculated by dividing the number of a certain category by the total live embryos at E18 in that basket (HH and HF) or tray (OH). Hatchability percentage was calculated by dividing the number of chickens that hatched per basket or tray by the number of live embryos at E18 of incubation.

### Day-Old Chicken Quality and Organ Weights

At D1, 40 chickens per pen were randomly selected for analysis of chicken quality indicators. Measurements included righting test, chicken length, body weight (including the residual yolk), scoring for navel, beak, and hock quality, and sex. First, chickens were subjected to a righting test, which was performed by putting the chicken on its back and measuring the time the chicken needed to stand up again, with a maximum of 20 s, as described by Rutkiewicz et al. (2013). Chicken length was measured from the tip of the beak to the tip of the middle toe (excluding the nail) by stretching the chicken along a ruler (Hill, 2001). After that, chickens were weighed. Then, navel condition was scored on a 3-point scale, with 1 = good (closed and clean navel area), 2 = moderate (black button up to 2 mm or black string), and 3 = poor quality (black button exceeding 2 mm or open navel area) (Molenaar et al., 2010). Beaks and hocks were scored on a 2-point scale (yes or no), with 1 = no red beak or hocks and 2 = red beak or hocks. Feather sexing was used to determine the sex of the chickens.

From the 40 originally selected chickens, 15 chickens were randomly selected for measurement of residual yolk and organ weights. Chickens were sacrificed by cervical dislocation and stored at −20°C. To determine organ weights, chickens were thawed and residual yolk was removed and weighed, after which organs (heart, liver, stomach, intestines [filled with feed], spleen, and bursa of Fabricius) were dissected and weighed. Yolk-free body mass (**YFBM**) was calculated as chicken weight minus residual yolk weight. All organ weights were expressed as percentage of YFBM. In addition, intestinal length was measured for each chicken.

### Performance

Feed and water intake were recorded at pen level during the whole experimental period. A random sample of 50 chickens per pen was individually weighed at D0, 7, 14, and 21. At D32 and D39, 75 chickens per pen were individually weighed. Owing to the different times of placement for the HH and HF chickens at D0, first the OH chickens were weighed (around 9:00 h), followed by the HH (around 11:00 h) and HF chickens (around 16:00 h). Mortality (number of chickens found dead) and culls (number of chickens euthanized because of compromised health or being extremely small) were recorded daily, and if known, the reason for the mortality was recorded. The FCR was calculated over the whole production period between D0 and D39 (FCR total). From the performance data, the European Production Efficiency Factor was calculated: **EPEF** = (1 - % mortality) × mean bird weight/mean length cycle/FCR total × 10.

### Slaughter Yield and Breast Myopathies

At D40 of age, just before slaughter, 36 chickens per pen (18 males and 18 females) were randomly selected, individually marked by a tag in both wings and weighed, and placed in marked transport containers, after which they were transported to a commercial processing plant together with all remaining chickens. The birds were slaughtered and eviscerated by trained slaughter plant personnel on an automated poultry processing line. Tagged carcasses were recovered from the processing line after plucking and stored at −20°C for further measurements of slaughter yield. Carcasses were thawed, and carcass yield (as a percentage of the live weight) and processing yields (as a percentage of the carcass weight) of the different commercial parts, (front half [includes breast, wings, skin, bones, small part of the back], back half [complete back half cut, including legs, part of the back, and skin], and breast, wings, and skin, separately), were determined. In addition, breast fillets (*Pectoralis major*) were submitted to visual and palpatory inspection for myopathies, including white striping (**WS**) and wooden breast (**WB**), using the scoring method of Kuttappan et al. (2016). Briefly, for WS, breast fillets were scored on a 4-point scale, with 0 = normal (no distinct white lines) and 3 = extreme (thick white bands > 2 mm thick); and for WB, breast fillets were scored on a 3-point scale from 0 (normal consistency) to 2 (very hard consistency).

### Statistical Analysis

All statistical analyses were performed using GenStat (version 19.1, VSN International). Differences with *P* < 0.05 were considered statistically significant, and 0.05≤*P* ≤ 0.10 were considered a trend. The measurements taken of individual chickens were aggregated per pen and per production cycle (for each combination of age or sex, if needed). The normality of the data was checked using residual plots. A natural log transformation of the aggregated measure was applied when variance was increased for increased levels of measures. The general model structure included the random effects of the nested design (sex [if applicable] within pen within room within production cycle). A room (containing 2 pens) within production cycle was the experimental unit for the main effect of hatching system (split-plot with room being the main plot and pen being the subplot). Hatchability of fertile eggs was calculated per treatment per production cycle and analyzed, using a general ANOVA.

For the chicken quality indicators and organ weights at D1, ANOVA was used to test for the fixed effects of hatching system, sex, and their interaction. Navel condition scores (3-point scale) were analyzed as ordinal variable, whereas red beak and red hock scores (2-point scale) were analyzed as binomial variables with a generalized linear model, using a logit link.

For measurements of BW, FCR, slaughter weight at D39, variation coefficient of BW at D39 and EPEF, a general ANOVA was used to test for the effects of hatching system. Measures of BW that were performed on different ages were analyzed using a mixed (REML) model with repeated measures to test for the fixed effects of hatching system, age, and their interaction. The BW were natural log transformed before testing. Predicted means (on a log scale) were back transformed to produce the estimated BW per age. Slaughter yield parameters were tested with ANOVA. The WS scores (4-point scale) and WB scores (3-point scale) were analyzed as ordinal variables with a generalized linear model, using a logit link.

## Results

### Hatchability of Fertile Eggs

Hatchability of fertile eggs on d 18 of incubation was on average 98.87 ± 0.34% for the HH system, 98.86 ± 0.34% for the HF system, and 98.97 ± 0.34% for the OH system (F_2,6_ = 0.03; *P* = 0.97). There was no difference between hatching systems in the causes or moments of embryo mortality (data not shown; all *P* > 0.05).

### Day-Old Chicken Quality and Organ Weights

Table 1 shows the chicken quality indicators and organ weights of HH, HF, and OH chickens at D1. For the indicators where a significant interaction between hatching system and sex was found, the results are presented in Table 2. Both HF and OH chickens were heavier than HH chickens (F_2,13_ = 47.96; *P* < 0.001), and HF and OH chickens also had a higher YFBM than HH chickens (F_2,13_ = 58.74; *P* < 0.001) (Table 1). Residual yolk weight did not differ between hatching systems (F_2,13_ = 0.18; *P* = 0.84; Table 1). The OH chickens had a higher, thus worse, navel score compared with both HH and HF chickens (Wald statistic = 29.77; *P* < 0.001). Time needed to stand up in the righting test was not affected by hatching system (Table 1). With respect to organ weights, relative weight of stomach was higher in the OH chickens than in the HH chickens, but HF did not differ from OH and HH (Wald statistic = 8.07; *P* = 0.03; Table 1). The OH chickens had a higher relative intestinal weight than HH chickens with HF chickens in between (Wald statistic = 19.34; *P* < 0.001) (Table 1). No hatching system effects were found for liver, spleen, and bursa weights (Table 1).

**Table 1:** Day-old chicken characteristics of hatchery-hatched (nonfed) (HH), hatchery-fed (HF), and on-farm hatched (OH) broiler chickens at D1 (LSmeans ± SEM). This table only includes the indicators where no hatching system∗sex interaction was found.

Chicken quality^1^	Hatching system				Sex			*P*-value		
	HH	HF	OH	SEM	Female	Male	SEM	P _system_	P _sex_	P_system∗ sex_
Righting test (s)	1.4	1.4	1.9	0.20	1.7^a^	1.5^b^	0.08	0.16	**0.04**	0.91
Chicken weight (g)	50.5^b^	57.5^a^	57.3^a^	0.58	55.7^a^	54.6^b^	0.25	**<0.001**	**0.004**	0.13
YFBM (g)	48.6^b^	55.7^a^	55.9^a^	0.54	54.0^a^	52.7^b^	0.40	**<0.001**	**0.03**	0.76
Residual yolk (g)	2.08	2.03	2.01	0.08	2.03	2.05	0.06	0.84	0.75	0.31
Navel condition (% per class)^2^								**<0.001**	**0.004**	0.56
1 (good)	46.3	40.9	28.2		35.0	42.0				
2 (moderate)	53.5	58.8	70.2		64.1	57.6				
3 (poor)	0.2	0.4	1.6		1.0	0.5				
Organ measurements^3^										
Liver weight (% of YFBM)	4.06	4.12	4.07	0.08	4.18^a^	3.99^b^	0.05	0.73	**<0.001**	0.17
Stomach weight (% of YFBM)	9.70^b^	9.95^a,b^	10.10^a^	0.06	9.83	10.00	0.11	**0.03**	0.12	0.10
Intestines weight (% of YFBM)	9.23^b^	10.11^b^	11.66^a^	0.23	10.46	10.22	0.40	**<0.001**	0.54	0.37
Spleen weight (% of YFBM)	0.039	0.040	0.039	0.001	0.036^a^	0.043^b^	0.002	1.00	**0.002**	0.35
Bursa weight (% of YFBM)	0.10	0.10	0.10	0.004	0.10^b^	0.11^a^	0.003	0.28	**<0.001**	0.52

^a,b^LSmeans within a row and factor lacking a common superscript differ (*P* < 0.05).

Bold indicates statistical significant differences (*P* < 0.05).

Abbreviation: YFBM: yolk-free body mass.

1 40 chickens per pen were selected for chick quality measurements.

2 Navel condition was analyzed within an ordinal scale model; therefore, no pairwise comparison for each class is presented.

3 15 chickens per pen were selected for organ measurements.

**Table 2:** Interactions between hatching system and sex on chicken length, intestinal length, relative heart weight, and hock scores of hatchery-hatched (nonfed) (HH), hatchery-fed (HF), and on-farm hatched (OH) broiler chicks at D1 (LSMeans±SEM).

Indicator^1^	HH female	HF female	OH female	HH male	HF male	OH male	SEM	P _system∗__sex_
Chicken length (cm)	20.0^a^	20.3^b^	20.6^c^	20.0^a^	20.3^b^	20.3^b^	0.05	**0.03**
Length of intestines (cm)	66.0^a^	75.1^c^	77.2^c^	66.2^a^	71.7^b^	78.4^c^	1.37	**0.04**
Heart weight (% of YFBM)	0.66^a,b^	0.64^a^	0.65^a,b^	0.68^a,b^	0.69^b,c^	0.72^c^	0.01	**0.05**
% Chicks with red hock	0.0^a^	0.4^a,b^	2.5^b,c^	0.9^b^	0.0^a^	8.2^c^		**0.005**

^a–c^LSmeans within a row lacking a common superscript differ (*P* < 0.05).

Bold indicates statistical significant differences (*P* < 0.05).

Abbreviation: YFBM: yolk-free body mass.

1 40 chickens per pen were selected for day-old chicken quality measurements; 15 out of 40 chickens per pen were selected for intestinal length and heart weight measurements.

Significant effects of sex were found for body weight with female day-old chickens being heavier than male chickens (F_1,33_ = 9.77; *P* = 0.004) and also having a higher YFBM than male chickens (F_1,33_ = 5.53; *P* = 0.03) (Table 1). In the righting test, female chickens took longer to stand up than male chickens (F_1,33_ = 4.81; *P* = 0.04). Female chickens had a worse navel score than male chickens (Wald statistic = 8.26; *P* = 0.004) (Table 1). Relative weight of liver (Wald statistic = 11.44; *P* < 0.001), spleen (Wald statistic = 10.16; *P* = 0.002), and bursa (Wald statistic = 11.58; *P* < 0.001) were also affected by sex, with females having a higher relative liver weight but a lower relative spleen and bursa weight than males (Table 1).

Chicken length showed an interaction between hatching system and sex (F_2,33_ = 3.92; *P* = 0.03); OH female chickens were longer than HH male and female chickens, with the HF chickens in between (Table 2). Hock score also showed an interaction between hatching system and sex (Wald statistic = 11.91; *P* = 0.005). The percentage of chickens with a red hock was lowest for the HH female chickens and HF male chickens (0% for both) and highest for OH male chickens (8.2%), with the other treatment groups in between (Table 2). Intestinal length did not differ between sexes in the HH and OH chickens, whereas in the HF chickens, females had longer intestines than males (interaction hatching system∗sex; Wald statistic = 6.49; *P* = 0.04; Table 2). In females, no effect of hatching system on relative heart weight was found, whereas in males, HH chickens had lower relative heart weight then OH chickens, with HF chickens in between (interaction hatching system∗sex; Wald statistic = 6.08; *P* = 0.05) (Table 2).

### Performance

Body weight development was affected by hatching system (Wald statistic = 319.96; *P* < 0.001), age (Wald statistic = 435,701.26; *P* < 0.001), and their interaction (Wald statistic = 161.74; *P* < 0.001). On D0, D7, D14, and D32, body weight was highest for OH followed by HF and HH. At D21 and D39, both OH and HF broilers were significantly heavier than HH broilers (Table 3). First wk mortality, total mortality, and the proportion of chickens found dead did not differ between hatching systems (Table 4). Total proportion of chickens culled was affected by system (F_2,13_ = 3.86; *P* = 0.048), with HF having a higher overall proportion total culled than HH and OH (Table 4). Cumulative feed intake (F_2,13_ = 22.31; *P* < 0.001), cumulative water intake (F_2,13_ = 6.07; *P* = 0.014), cumulative water–feed ratio (F_2,13_ = 11.61; *P* = 0.001), and variation coefficient of BW at D39 (F_2,13_ = 6.58; *P* = 0.011) were affected by hatching system. Cumulative feed and water intake were higher for OH chickens compared with HH and HF chickens. The cumulative water–feed ratio was higher for HH chickens compared with both HF and OH chickens. The variation coefficient of BW at D39 was lower for OH chickens compared with both HH and HF chickens. The EPEF tended to be higher for the OH than for the HH treatment, with the HF treatment in between. No treatment differences were found for FCR (Table 4).

**Table 3:** Predicted means for body weight ± SEM of hatchery-hatched (nonfed) (HH), hatchery-fed (HF), and on-farm hatched (OH) broiler chickens between D0 and D39.

Body weight (g)^1^	Hatching system		
	HH	HF	OH
Day 0	36.9 ± 0.5^c^	43.4 ± 0.5^b^	46.6 ± 1.4^a^
Day 7	167.0 ± 6.7^c^	182.3 ± 6.2^b^	188.4 ± 3.2^a^
Day 14	481 ± 18^c^	510 ± 18^b^	530 ± 14^a^
Day 21	971 ± 31^b^	1,026 ± 37^a^	1,045 ± 37^a^
Day 32	1,944 ± 81^c^	2,025 ± 84^b^	2,082 ± 45^a^
Day 39	2,634 ± 88^b^	2,718 ± 78^a^	2,750 ± 75^a^
P-value			
P _system_	**<0.001**		
P _age_	**<0.001**		
P _system∗age_	**<0.001**		

^a–c^LSmeans within a row lacking a common superscript differ (*P* < 0.05).

Bold indicates statistical significant differences (*P* < 0.05).

1 Body weights based on a sample of 50 broilers per pen, apart from day 32 and day 39, when 75 chickens were weighed.

**Table 4:** Predicted means ± SEM for performance indicators of hatchery-hatched (nonfed) (HH), hatchery-fed (HF), and on-farm hatched (OH) broiler chickens over the whole rearing period (D0-39).

Indicator^1^	Hatching system				P _system_
	HH	HF	OH	SEM	
First wk mortality (%)	1.83	2.43	1.59	0.39	0.32
Total mortality (%)^2^	3.27	4.39	3.20	0.47	0.17
Total found dead (%)^2^	1.88	2.09	1.62	0.25	0.45
Total culled (%)^2^	1.39^b^	2.30^a^	1.57^b^	0.25	**0.05**
Cumulative feed intake (g/chicken)	3,823^c^	3,962^b^	4,045^a^	23.8	**<0.001**
FCR total^3^	1.46	1.46	1.48	0.009	0.28
Cumulative water intake (ml/chicken)	7,346^b^	7,323^b^	7,477^a^	33.7	**0.01**
Cumulative water-feed ratio	1.92^a^	1.84^b^	1.83^b^	0.013	**0.001**
Variation coefficient of body weight at D39 (%)	12.7^a^	13.1^a^	11.6^b^	0.3	**0.01**
EPEF^4^	426.9	434.8	442.0	4.3	0.08

^a–c^LSmeans within a row lacking a common superscript differ (*P* < 0.05).

Bold indicates statistical significant differences (*P* < 0.05).

1 FCR total, variation coefficient of BW at D39, and EPEF are based on BW of 75 chickens per pen.

2 Total mortality is the sum of total culled and total found dead. Total found dead represents all chickens that were found dead during inspection of the pens; total culled represents the proportion of chickens that were euthanized because of compromised health or being extremely small.

3 FCR total: feed conversion ratio calculated over the whole production period between D0 and D39.

4 European Production Efficiency Factor (EPEF) = (1 - % mortality) × mean bird weight/mean length cycle/net feed conversion × 10.

### Slaughter Yield and Breast Myopathies

Live body weight (F_2,13_ = 21.57; *P* < 0.001) and carcass weight (F_2,13_ = 16.83; *P* < 0.001) at slaughter were highest for OH chickens, followed by HF and HH chickens. Wings yield (F_2,13_ = 7.68; *P* = 0.006) was higher for HH chickens compared with both HF and OH chickens (Table 5). The OH and HF chickens had higher, thus worse, WB and WS scores compared with HH chickens (WB: Wald statistic = 6.46; *P* = 0.04; WS: Wald statistic = 12.76; *P* = 0.002) (Table 6).

**Table 5:** Carcass yield of hatchery-hatched (nonfed) (HH), hatchery-fed (HF), and on-farm hatched (OH) broiler chickens at slaughter (D40) (LSmeans ±SEM).

Carcass yield^1^	Hatching system				Sex			*P*-value		
	HH	HF	OH	SEM	Female	Male	SEM	P _system_	P _sex_	P _system∗sex_
Live weight (g)	2,689^c^	2,772^b^	2,854^a^	18.0	2,558^b^	2,985^a^	13.0	**<0.001**	**<0.001**	0.58
Carcass weight (g)	1,844^c^	1,902^b^	1,963^a^	15.0	1,766^b^	2,042^a^	9.6	**<0.001**	**<0.001**	0.53
Carcass (% of live weight)	68.8	68.6	69.1	0.3	69.1^a^	68.5^b^	0.1	0.42	**<0.001**	0.86
Front half (% of carcass weight)^2^	56.9	57.1	57.4	0.2	57.4^a^	56.9^b^	0.1	0.26	**0.03**	0.19
Breast (% of carcass weight)	41.6	42.1	43.0	0.5	42.6	41.8	0.4	0.19	0.20	0.38
Wings (% of carcass weight)	11.0^a^	10.8^b^	10.8^b^	0.07	10.9^a^	10.8^b^	0.05	**0.006**	**0.004**	0.32
Skin (% of carcass weight)	4.6	4.4	4.6	0.1	4.6^a^	4.5^b^	0.04	0.23	**0.005**	0.92
Back half (% of carcass weight)^3^	42.9	42.6	42.6	0.3	42.5^b^	42.9^a^	0.12	0.43	**0.001**	0.95

^a–c^LSmeans within a row and factor lacking a common superscript differ (*P* < 0.05).

Bold indicates statistical significant differences (*P* < 0.05).

1 36 chickens (18 males plus 18 females) per pen were selected for these measurements.

2 Front half: includes breast, wings, skin, bone, small part of the back (neck was sometimes partially present but excluded from the front half weight).

3 Back half: complete back half cut, including legs, part of the back and skin.

**Table 6:** Distribution of hatchery-hatched (nonfed) (HH), hatchery-fed (HF), and on-farm hatched (OH) broiler chickens within the different wooden breast (WB) and white striping (WS) scoring categories measured in *Pectoralis major* muscles at slaughter.

Breast myopathies	Hatching system			Sex		*P*-value		
	HH	HF	OH	Female	Male	P _system_	P _sex_	P _system∗sex_
Distribution (%) of WB scores in the total sample^1^								
0 = normal consistency	24.56	16.07	15.60	28.27	9.21	**0.04**	**<0.001**	0.51
1 = hard consistency	34.72	35.76	36.97	38.90	32.13			
2 = very hard consistency	40.70	48.17	48.33	32.83	58.66			
Distribution (%) of WS scores in the total sample^1^								
0 = normal, no distinct white lines	9.81	5.14	5.22	9.72	3.72	**0.002**	**<0.001**	0.32
1 = moderate, small white lines (<1 mm thick)	37.02	31.53	21.61	33.31	26.80			
2 = severe, large white lines (1–2 mm thick)	48.87	57.69	66.86	52.26	63.35			
3 = extreme, thick white bands (>2 mm thick)	4.30	5.64	6.31	4.71	6.13			

^a,b^LSmeans within a row and factor lacking a common superscript differ (*P* < 0.05).

Bold indicates statistical significant differences (*P* < 0.05).

1 WB and WS scores were analyzed within an ordinal scale model; therefore, no pairwise comparison for each class is presented; 36 chickens (18 males plus 18 females) per pen were selected for these measurements.

Except for breast yield, all slaughter yield variables were affected by sex (Table 5). Male chickens were heavier than female chickens at slaughter (F_1,33_ = 535.01; *P* < 0.001) and had heavier carcasses than female chickens (F_1,33_ = 413.63; *P* < 0.001). However, carcass yield (F_1,33_ = 18.99; *P* < 0.001), front half yield (F_1,33_ = 5.12; *P* = 0.03), wings yield (F_1,33_ = 9.87; *P* = 0.004), and skin yield (F_1,33_ = 9.12; *P* = 0.005) were higher for female chickens than for male chickens. Back half yield was higher for male chickens than for female chickens (F_1,33_ = 12.78; *P* = 0.001). The WB and WS scores were also affected by sex (Table 6). Male broilers had higher thus worse WB and WS scores than female broilers (WB: Wald statistic = 91.82; *P* < 0.001; WS: Wald statistic = 30.39; *P* < 0.001).

## Discussion

The present study showed that in chickens from young parent stock flocks, body weight and development of day-old hatchery-fed or on-farm hatched chickens was better compared with conventionally hatched chickens. On-farm hatched chickens had worse navel and hock quality at D1, but this did not seem to result in any negative effects on later performance. Both on-farm hatching and early feeding in the hatchery had a long-term positive effect on body weight. However, also a higher prevalence of breast myopathies at slaughter age in on-farm hatched and hatchery-fed chickens was found compared with conventionally hatched chickens.

### Day-Old Chicken Quality

In the current study, it was shown that hatchability of fertile eggs was similar between hatching systems, and this may indicate that variations in environmental conditions within certain limits during the perihatching phase does not seem to affect survival of broiler embryos, despite different air speeds and settings of temperature and relative humidity and different CO_2_ concentration surrounding the eggs in the different hatching systems.

In accordance with previous results (de Jong et al., 2019, 2020), day-old OH and HF chickens were heavier than HH chickens, likely because they could eat and drink immediately after hatching (van de Ven et al., 2009). In accordance with this, day-old chickens were longer for HF and OH than HH, suggesting a better day-old chicken quality and possibly also a better posthatch performance, although the relationship between chicken length and later life performance does not always seem to be very strong (Willemsen et al., 2008). In the current study, chickens hatched on average at E20 (unpublished data), which means that the fed chickens were on average 36 h longer on feed and water and were able to start their development sooner than the HH chickens. On-farm hatching and hatchery-fed chickens had a higher stomach and intestinal weight than the hatchery-hatched chickens, likely because of an earlier onset of feed intake and development. Longer intestines were found for OH (male) chickens compared with HH and HF chickens. Gastrointestinal development is stimulated by the intake of feed after hatching (Jin et al., 1998). The OH chickens seemed to have the largest advantage in the early development of their gastrointestinal tract as indicated by longer intestines. The HF chickens had longer intestines than HH chickens as well, but they were shorter than intestines of OH chickens. The difference between OH and HF in gastrointestinal development might be related to the composition of the prestarter diet provided to HF chickens in the hatchery as part of the HF system, but posthatch events also may have played a role. Although HF chickens could eat and drink in the hatcher, they still had to be handled and transported to the broiler farm, during which they were not provided with water. Transportation will probably result in lower feed consumption and may also induce stress (Sun et al., 2018), which may slow down organ development. According to Lamot et al. (2014), even a short delay of less than 24 h in feed access after hatching can decrease growth and intestinal development during the first 4 d after hatching. This is often compensated in the long term through compensatory growth, although in the present study at slaughter OH and HF still differed in body weight.

Other indicators of day-old chicken quality, that is, navel condition and red hocks (van de Ven et al., 2012), were worse for OH chickens compared with the other treatments. This is in accordance with previous findings (de Jong et al., 2019, 2020), and it could be because of suboptimal or more variation in hatching conditions at the broiler farm. It has been found that a high incubation temperature and consequently larger residual yolk can be related to a worse navel quality, although other factors seem to be related as well (Molenaar et al., 2010; Nangsuay et al., 2016; Van den Brand et al., 2019). Furthermore, there might have been differences in the selection of second grade chickens between the hatching systems. This is because the removal of second grade chickens was performed by animal caretakers in the OH treatment, which could have been less strict compared with the selection performed by experienced personnel in the hatchery for the HH and HF treatments. In all 3 production cycles, it was found that the percentage of second grade chickens was lower in the OH compared with the HH and HF treatment (unpublished data). It might be more difficult to select second grade chickens if they are already on the floor compared with the higher and well-illuminated conveyor belt in the hatchery. A poor navel quality has been associated with a lower survival and lower posthatch growth (Fasenko and O'Dea, 2008), although in the current study first wk and total mortality were not higher in OH flocks, confirming previous studies comparing HH and OH flocks (de Jong et al., 2019, 2020). This could mean that chicken quality measured by navel and hock quality was not sufficiently affected to alter performance of broiler chickens in later life or that navel and hock quality do not always relate to later performance.

Besides the differences between hatching systems, there were also significant differences between sexes. Female chickens were heavier and had higher YFBM than male chickens at D1. Van de Ven et al. (2011) did not find sex effects on growth between d 0 and 7, but it was found that female chickens hatched earlier than male chickens (Burke, 1992; Reis et al., 1997; van de Ven et al., 2011). Assuming that in the current study females also hatched earlier than males, it can be expected that females started earlier with feed intake and consequently were heavier at D1 than males. In the righting test, female chickens took longer to stand up than male chickens at D1. Righting times in our study ranged from 1 to 20 s, with the majority of chickens standing within 2 s, which is in accordance to previous findings of Rutkiewicz et al. (2013). It could be that heavier female chickens have more difficulty to stand up than lighter male chickens at D1, but the reason for sex-related differences in righting time remains to be investigated.

### Performance

During the rearing period, HF chickens had a higher total proportion of culled chickens compared with HH and OH chickens, but treatments did not differ significantly in both first wk and total mortality. Very small chickens were a major reason for culling, which may be explained by a lower flock uniformity as a result of the spread in hatch time and the onset of water and feed intake. On the other hand, a higher proportion of culled chickens was not observed in the OH treatment, and they showed even a better, thus lower, variation coefficient of body weight at D39 compared with the HF treatment. Possibly, fed chickens are more susceptible to stress before and during transportation compared with unfed chickens. There is limited information about the effect of transport of fed chicks on performance. In nonfed chickens, Bergoug et al. (2013) found a negative effect of transport on body weight until 21 d of age, that is chickens that were transported during 4 and 10 h had lower body weights than chickens that were not transported. Thus, perhaps a combination of factors (e.g., parent stock age, gut filling, holding time, and transportation) might have negatively affected the development of the HF chickens and have resulted in increased culling. Nevertheless, the exact reason of the higher culling in HF chickens remains to be further investigated.

From D0 onward, body weight of OH and HF chickens was significantly higher than HH chickens, and this difference was observed until slaughter age. This is in accordance with the review of de Jong et al. (2017), where a meta-analysis of various studies under controlled conditions showed that immediate access to feed and water after hatching resulted in higher body weight at slaughter age compared with prolonged duration of feed and water deprivation (≥36 h). However, they also showed that there is variation between studies, with some studies reporting only short-term effects of early feeding on body weight (van de Ven et al., 2011, Hollemans et al., 2018; de Jong et al., 2019, 2020). Effects on body weight seemed to be stronger in OH than in HF chickens, with OH being significantly heavier than HF between D0 to 14, D32, and at slaughter (D40). Possibly, the lack of stressful events after hatching, such as handling and transport in OH chickens compared with HF chickens (Bergoug et al., 2013; Hollemans et al., 2018), played a role. Further, OH chickens spent longer time in their home pens with feed and water available *ad libitum*, while HF chickens had to be transported from the hatchery, resulting in disrupted feed intake. In comparison to previous studies, where a short-term effect of on-farm hatching was found on body weight (de Jong et al., 2019, 2020), in the current study, a different pattern was found, that is higher body weight in the early fed chickens was found from D0 onward until slaughter age. This long-term higher body weight in HF and OH broilers could be specific for chickens of young parent stock. These chickens are usually smaller and more sensitive for suboptimal conditions (Weyntjens et al., 1999) and may benefit more from early feeding until slaughter age. Further, as indicated earlier, chickens hatched at E20, which means that the posthatch feed deprivation for HH was on average 36 h. A shorter posthatch feed deprivation might not have resulted in these long-term differences in body weight between the treatments (de Jong et al., 2017).

### Slaughter Yield

In accordance with the body weight differences between D0 and D40, OH had higher carcass weights than HF, followed by HH. However, both HF and OH chickens had worse WB and WS scores at slaughter compared with HH chickens. The WB and WS are muscle myopathies observed in fast-growing broiler chickens (Barbut, 2019). The WB often appears together with WS (Sihvo et al., 2014), and there seems to be a relationship between fast growth rate and the occurrence of these breast myopathies in broilers (Kuttappan et al., 2013; Barbut, 2019). Thus, especially heavier chickens with high breast muscle yield and faster growth have consistently demonstrated higher WB and WS scores (Kuttappan et al., 2012, 2013, 2017), and this also seems to be the case for the OH and HF broilers. Males had higher prevalence of WB and WS than females, which is likely associated with the higher live body weight and carcass weight of male chickens (Barbut, 2019). Apart from carcass weight, carcass and processing yields were not affected by the treatments, except for wings yield, which was significantly higher for HH than for HF and OH, although the reason for this is unknown. Not surprisingly, males had heavier live body weight at slaughter and heavier carcass weights than females, and these might be related to the sex differences found for carcass and processing yields (e.g., Hussein et al., 2019).

## Conclusions

In conclusion, both on-farm hatching and hatchery feeding were beneficial for day-old chicken development and performance in chickens from young parent stock flock, with on-farm hatching showing better overall performance than hatchery feeding. The worse day-old chicken quality of on-farm hatched chickens compared with the other treatments (measured by navel and hock condition) did not affect first wk mortality and later life performance. However, we observed a higher prevalence of breast myopathies and thus worse meat quality, in both on-farm hatched and hatchery-fed broiler chickens compared with conventionally hatched chickens from young parent stock flocks, which could be related to the higher body weight of these treatments during the whole rearing period.
